# Diagnosing Infectious Hepatic Cysts in an Older Patient With Multiple Skin Masses: A Case Report

**DOI:** 10.7759/cureus.35993

**Published:** 2023-03-10

**Authors:** Kotaro Murakami, Nozomi Nishikura, Tsuyoshi Mishiro, Chiaki Sano, Ryuichi Ohta

**Affiliations:** 1 Postgraduate Clinical Training Center, Shimane University Hospital, Izumo, JPN; 2 Community Care, Unnan City Hospital, Unnan, JPN; 3 Internal Medicine, Unnan City Hospital, Unnan, JPN; 4 Community Medicine Management, Shimane University Faculty of Medicine, Izumo, JPN

**Keywords:** lymphoma, abdominal ultrasound, infectious hepatic cysts, rural hospital, general medicine

## Abstract

Among the most severe complications of hepatic cystic diseases is infectious hepatic cysts (IHCs). IHCs may appear mainly among immunocompromised hosts. However, older patients have a variety of immunological conditions. The detection of the factors suppressing immunity is essential for patients with IHCs. Herein, we present the case of an 86-year-old woman admitted to the emergency department with a fever. We suspected IHCs based on changes in abdominal ultrasound findings. After multiple follow-ups to determine the cause of the fever that was unresponsive to treatment, we discovered debris in the cyst that had not been present at the time of the initial presentation. The patient was subsequently treated with percutaneous transhepatic drainage and tazobactam/piperacillin. The investigation of the causes of immunosuppression clarified the multiple skin masses. The biopsy of the mass clarified diffuse large B cell lymphoma without lymph node swellings. Consecutive ultrasound can detect findings missed during the initial presentation and changes that occur within a short period. The detection of the causes of immunosuppression is essential even among older patients with IHCs for better care among older patients.

## Introduction

One of the most severe complications of hepatic cystic diseases is infectious hepatic cysts (IHCs). Hepatic cysts affect approximately 5% of the population; however, their prevalence varies [1]. IHCs are uncommon complications of hepatic cysts; however, the prevalence of IHCs may be underestimated [2]. The symptoms of IHC include fever, chills, and abdominal pain, similar to those of other infections that cause sepsis. The route of infection may be the biliary tract or bloodstream; however, in most cases, the route is unknown [2]. Causative microorganisms can be identified in 50% of IHCs, and intestinal bacteria are the leading cause of the disease [2,3]. Imaging studies are useful for approximately 70% of IHCs, and abdominal ultrasound, computed tomography (CT), magnetic resonance imaging (MRI), single-photon emission CT, and fluorodeoxyglucose-positron emission tomography (FDG-PET) are used for diagnosis [3].

Most IHCs are diagnosed through continuous follow-ups for fevers of unknown origin [4]. Many patients do not improve after treatment with antimicrobials alone, necessitating aggressive drainage [3,5]. Herein, we present the case of an 86-year-old woman admitted to the emergency department with a fever. We suspected IHCs based on changes in the abdominal ultrasound findings. We detected debris in the cyst that was not present at the time of the initial presentation. The patient was subsequently treated with percutaneous transhepatic drainage and antibiotics. We report on a case in which abdominal ultrasound findings contributed to the IHC diagnosis.

## Case presentation

An 86-year-old woman presented at a rural community hospital with the chief complaint of fever and loss of appetite. The patients presented with a similar chief complaint and had been treated for urinary tract infection one month prior to presentation. The day before the visit, she experienced fatigue; however, she could eat. On the morning of the day of the presentation, she could not eat and had a fever of 38.8°C and a headache; subsequently, she presented at the emergency department of the hospital. The patient had a history of giant hepatic cysts, hypertension, dyslipidemia, and gastroesophageal reflux disease. The giant hepatic cyst was a simple cyst that a gastroenterologist followed up on. Her medical history included the administration of betahistine mesylate, pravastatin sodium, esomeprazole, and magnesium hydrate.

Her vital signs at admission were as follows: temperature, 38.8°C; pulse rate, 73 beats/min; blood pressure, 145/80 mmHg; respiratory rate, 24 breaths/min; and SPO2, 91% (room air). Multiple pedicled-sized masses were observed on the abdominal wall; however, no other physical findings, including Murphy’s sign, percussion tenderness on the costovertebral angles. Blood tests were normal except for a mildly reduced platelet count (Table 1).

**Table 1:** Laboratory data at the initial presentation WBC: white blood cells; RBC: red blood cells; Hb: hemoglobin; Hct: hematocrit; MCV: mean corpuscular volume; Plt: platelet; TP: total protein; ALB: albumin; total bill: total bilirubin; AST: aspartate aminotransferase; ALT: alanine aminotransferase; ALP: alkaline phosphatase; GGT: γ-glutamyl transpeptidase; LDH: lactate dehydrogenase; Ig: immunoglobulin

Parameter	Value	Reference
WBC	6.9	3.5-9.8×10^³^/μL
RBC	4.92	4.10-5.30×10^⁶^/μL
Hb	12.8	13.5-17.6 g/dL
Hct	37.5	36-48%
MCV	95.6	82-101 fL
Plt	11.6	13.0-36.9×10^⁴^/μL
TP	7.3	6.6-8.1 g/dL
ALB	2.8	3.9-4.9 g/dL
Total Bil	1.0	0.2-1.2 mg/dL
AST	26	8-38 IU/L
ALT	9	4-44 IU/L
ALP	132	38～113 U/L
GGT	18	16-73 IU/L
LDH	221	106-211 U/L
IgG	2019	870-1700 mg/dL
IgA	259	110-410 mg/dL
IgE	15	<173 IU/mL
IgM	106	33-190 mg/dL
Urine analysis		
WBC	Positive	Negative
Nitrite	Negative	Negative
Protein	Positive	Negative
Glucose	Negative	Negative
Urobilinogen	Positive	Negative
Bilirubin	Negative	Negative
Ketone bodies	Negative	Negative
Blood	Negative	Negative
pH	7.5	
Specific gravity	1.022	

**Figure 1:**
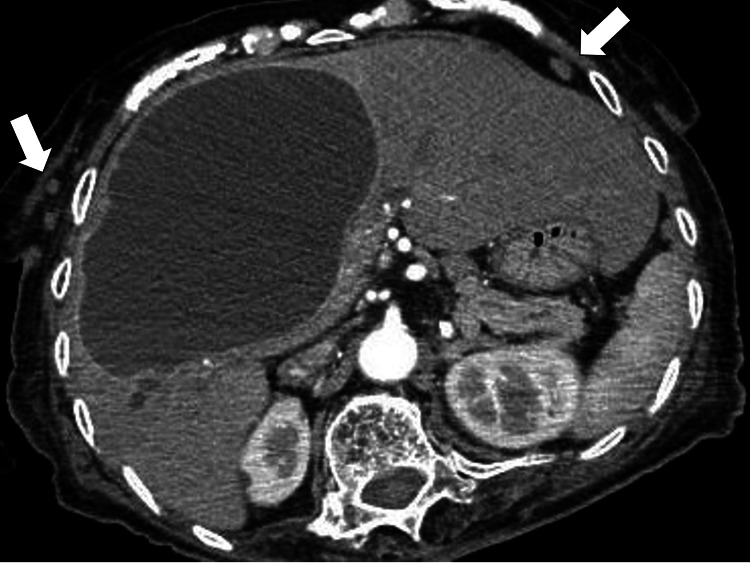
Contrast-enhanced computed tomography showing multiple masses on the chest wall (white arrows) and no mosaic density changes in the intrahepatic cyst

Abdominal ultrasonography revealed a giant cyst in the liver with homogenous hypoechoic content. Abdominal CT showed multiple well-defined limbal masses in the abdominal wall and a hepatic cyst. Gram staining of the urine showed the presence of gram-negative rods. Based on these findings, we diagnosed the patient with a urinary tract infection and treated her with cefmetazole (4g/day). However, the fever did not resolve even after antibiotic administration; therefore, on day 4 of admission, contrast-enhanced abdominal and pelvic CT scans were performed, showing multiple masses on the chest wall (white arrows) and no mosaic density changes in the intrahepatic cyst (Figure 1).

**Figure 2:**
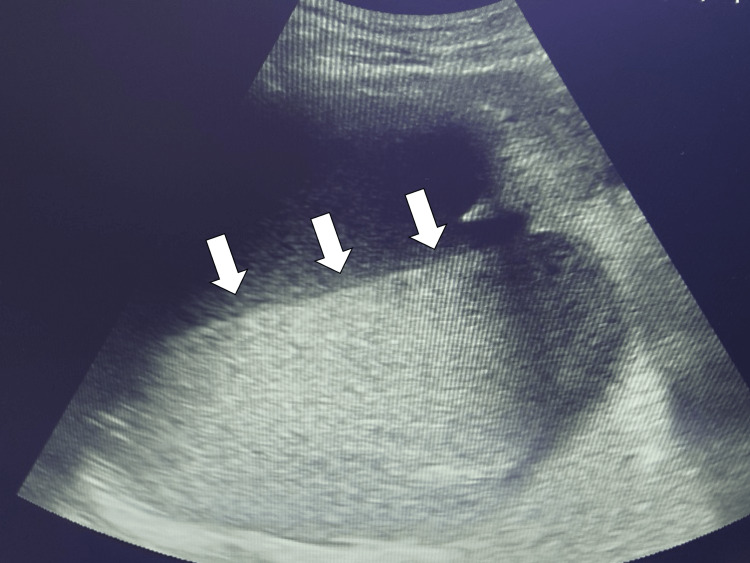
Abdominal ultrasound imaging showing debris in the hepatic cyst

On day 4 of admission, we biopsied the mass on the abdominal wall and made a pathological diagnosis of diffuse large B cell lymphoma (DLBCL), showing the immunosuppressive condition of the patient. Through the discussion with the patient and families, we started prednisolone of 15mg/day as palliative care. On day 7 of admission, her fever persisted at 38°C, and the abdominal ultrasound showed debris in the hepatic cyst (Figure 2).

**Figure 3:**
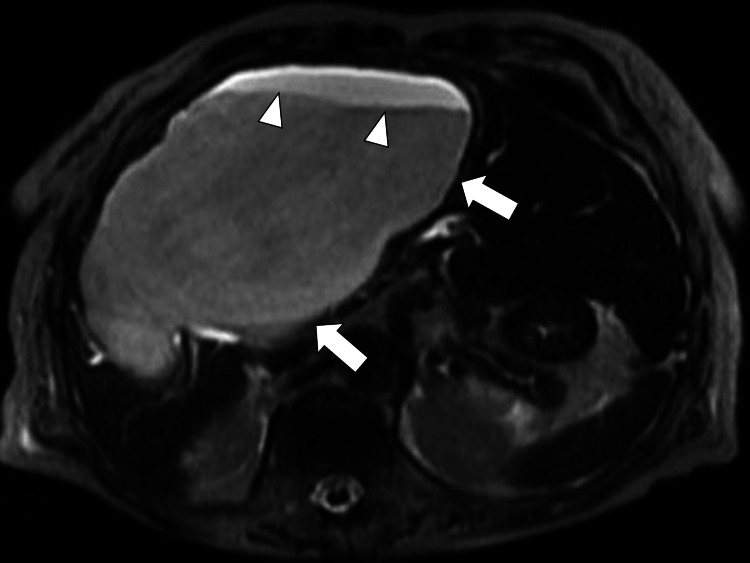
T2-weighted magnetic resonance imaging showing the hepatic cyst (white arrows) and debris (white arrowheads)

Debris was identifiable on the MRI (Figure 3).

We suspected IHC and performed a hepatic cyst puncture to obtain purulent puncture fluid. Gram staining of purulent fluid revealed the presence of gram-negative rods. On day 8 of admission, the blood culture of the previous day was positive for *Enterobacter cloacae*. The patient was diagnosed with IHCs, and drainage of the cyst was noted. The antibiotic was changed to piperacillin/tazobactam (13.5g/day) based on a drug sensitivity test of *E. cloacae*. The patient completed the antibiotic treatment on day 14 after drainage initiation, after confirming the shrinkage of the abscess using abdominal ultrasound. On day 16, the drainage tube was removed. There was no enlargement of the hepatic cysts, but the patient continued to have night sweats and a low-grade fever. The patient showed worsening of the nodules throughout the body due to the exacerbation of DLBCL, which was considered to be the cause of her symptoms. The patient was provided palliative care. On day 42, after admission, the patient died.

## Discussion

In our case, we diagnosed an older patient with DLBCL and IHCs using consecutive ultrasonography. IHCs are more common in patients with autosomal dominant polycystic kidney disease [3]. Furthermore, 30% of the patients with IHCs had a history of organ transplantation. Therefore, immunological conditions can affect the IHC results. Herein, our patient was immunocompromised with DLBCL, which impinged the immunity and was susceptible to developing IHCs.

Imaging studies should be performed consecutively and several times for patients suspected of IHCs. Approximately 78% of IHCs were suspected by imaging studies [3]. Of these, 43% were suspected of using FDG-PET/CT, 33% of CT, and 14% of abdominal ultrasound [3]. The imaging findings of the IHCs were based on those of pyogenic liver abscesses. A low absorption area with an internal septum, rim enhancement, and internal gas characterizes contrast-enhanced CT. Abdominal ultrasound findings showed various degrees of internal echogenicity, from low to high, and the appearance of debris [6]. CT (sensitivity, 97%) is more sensitive and helpful in detecting liver abscesses than ultrasonography (sensitivity, 85%) [7,8]. Gas images in the abscess cavity on CT have been observed in a few cases (9.6-18.5%) [7,9]. Rim enhancement varies among studies, ranging from 6% to 43.8% in pyogenic liver abscesses [10,11]. Septal enhancement is seen in 58.9% of patients, double target sign in 24.2%, and debris in 36.1% [10,11].

Abdominal ultrasound reveals abscesses in 60.4% of cases depending on examiners' skills, and when combined with equivocal findings, 85.8% of the cases are suspected to be IHCs [12]. A follow-up ultrasound revealed intracystic debris, which led to the puncture and diagnosis. Therefore, contrast-enhanced CT is valid for the diagnosis of IHCs. However, repeated imaging is associated with contrast-induced nephropathy and radiation exposure. Abdominal ultrasonography is simple, noninvasive, and can quickly be performed in the hospital ward. Therefore, abdominal ultrasound is a more suitable imaging modality for short intervals than contrast-enhanced CT [10]. Although ultrasonography is less sensitive than contrast-enhanced CT for IHCs, it is highly sensitive [11]. As our case shows, strenuous follow-up of the findings of hepatic cysts that were not present at the initial presentation may be more beneficial in progressively worsening cases.

The diagnosis of IHCs indicates immunocompromised conditions in previously immunocompetent older patients. In our case, the patient was old but had no complications or past medical histories suppressing her immunity. The investigation for diseases suppressing immunity clarified the presence of DLBCL. Common immunosuppressive conditions are diabetes and using steroids or immunosuppressive drugs [7]. However, among older patients, the presence of occult malignancy can be a cause of immunosuppression [8]. Older patients have a high risk of cancer, but their symptoms from cancers are vague and difficult to diagnose initially. Like our case, intravascular lymphoma and DLBCL can be challenging when patients do not have typical symptoms such as neck lymph node swelling and night sweats [10]. This patient had night sweats and low-grade fever during admission. The timing of diagnosing IHCs can also be critical timing investigating the presence of immunosuppressive diseases, including cancers.

The diagnosis and treatments of IHCs need system-specific management among older patients. Especially in rural contexts, older patients are followed by family physicians. Our patient was admitted to the rural community hospital, managed mainly by family physicians. Smooth management of older patients with complicated diseases such as IHCs should be managed systematically [13]. In rural contexts, rural family physicians should specialize in patients' systems and facilitate their quality of life (QOL), including palliative care, to effectively manage older immunosuppressive patients.

## Conclusions

We diagnosed IHCs using repeated abdominal ultrasound examinations for fever that could not be diagnosed at the initial presentation. Repeated abdominal ultrasonography is a useful imaging modality. Furthermore, older patients with IHCs should be investigated for immunosuppressive diseases such as lymphoma.

## References

[1] Asuquo M, Nwagbara V, Agbor C, Otobo F, Omotoso A (2015). Giant simple hepatic cyst: a case report and review of relevant literature. Afr Health Sci.

[2] Morii K, Yamamoto T, Nakamura S, Okushin H (2018). Infectious hepatic cyst: an underestimated complication. Intern Med.

[3] Lantinga MA, Geudens A, Gevers TJ, Drenth JP (2015). Systematic review: the management of hepatic cyst infection. Aliment Pharmacol Ther.

[4] Ikeda H, Ohta R, Nishikura N, Ryu Y, Sano C (2022). The persistent approach to diagnose infectious hepatic cysts in a patient with recurrent fever: a case report. Cureus.

[5] Mori E, Akai Y, Matsumoto T (2012). Hepatic cyst infection in a healthy older male. BMJ Case Rep.

[6] Mortelé KJ, Segatto E, Ros PR (2004). The infected liver: radiologic-pathologic correlation. Radiographics.

[7] Serraino C, Elia C, Bracco C (2018). Characteristics and management of pyogenic liver abscess: a European experience. Medicine (Baltimore).

[8] Yin D, Ji C, Zhang S (2021). Clinical characteristics and management of 1572 patients with pyogenic liver abscess: a 12-year retrospective study. Liver Int.

[9] Khim G, Em S, Mo S, Townell N (2019). Liver abscess: diagnostic and management issues found in the low resource setting. Br Med Bull.

[10] Halvorsen RA, Korobkin M, Foster WL, Silverman PM, Thompson WM (1984). The variable CT appearance of hepatic abscesses. AJR Am J Roentgenol.

[11] Lee JH, Jang YR, Ahn SJ, Choi SJ, Kim HS (2020). A retrospective study of pyogenic liver abscess caused primarily by Klebsiella pneumoniae vs. non-Klebsiella pneumoniae: CT and clinical differentiation. Abdom Radiol (NY).

[12] Lin AC, Yeh DY, Hsu YH, Wu CC, Chang H, Jang TN, Huang CH (2009). Diagnosis of pyogenic liver abscess by abdominal ultrasonography in the emergency department. Emerg Med J.

[13] Ohta R, Sano C (2022). Family physicians as system-specific specialists in Japan’s aging society. Cureus.

